# AKR1C2 acts as a targetable oncogene in esophageal squamous cell carcinoma via activating PI3K/AKT signaling pathway

**DOI:** 10.1111/jcmm.15604

**Published:** 2020-07-17

**Authors:** Zhan‐Fei Zhang, Tie‐Jun Huang, Xin‐Ke Zhang, Yu‐Jie Xie, Si‐Ting Lin, Fei‐Fei Luo, Dong‐Fang Meng, Hao Hu, Jing Wang, Li‐Xia Peng, Chao‐Nan Qian, Chao Cheng, Bi‐Jun Huang

**Affiliations:** ^1^ Department of Thoracic Surgery The First Affiliated Hospital of Sun Yat‐Sen University Guangzhou China; ^2^ Department of Experimental Research State Key Laboratory of Oncology in South China Collaborative Innovation Center for Cancer Medicine Sun Yat‐Sen University Cancer Center Guangzhou China; ^3^ Department of Nuclear Medicine The Second People's Hospital of Shenzhen Shenzhen China; ^4^ Department of Nasopharyngeal Carcinoma Sun Yat‐Sen University Cancer Center Guangzhou China; ^5^ Department of Pathology Sun Yat‐Sen University Cancer Center Guangzhou China; ^6^ Department of Thoracic Surgery The People's Hospital of Gaozhou Maoming China; ^7^ Department of Traditional Chinese Medicine The First Affiliated Hospital of Sun Yat‐Sen University Guangzhou China; ^8^ Department of Radiation Oncology Guangzhou Concord Cancer Center Guangzhou China

**Keywords:** AKR1C2, cisplatin resistance, combination therapy, oesophageal squamous cell carcinoma, PI3K/AKT signalling pathway

## Abstract

The aldo‐keto reductases family 1 member C2 (AKR1C2) has critical roles in the tumorigenesis and progression of malignant tumours. However, it was also discovered to have ambiguous functions in multiple cancers and till present, its clinical significance and molecular mechanism in oesophageal squamous cell carcinoma (ESCC) has been unclear. The aim of this study was to explore the role of AKR1C2 in the tumorigenesis of ESCC. Here, we showed that AKR1C2 expression was found to be up‐regulated in ESCC tissues and was significantly associated with pathological stage, lymph node metastasis and worse outcomes. Functional assays demonstrated that an ectopic expression of AKR1C2 in ESCC cells resulted in increased proliferation, migration and cisplatin resistance, while knockdown led to inversing effects. Bioinformation analyses and mechanistic studies demonstrated that AKR1C2 activated the PI3K/AKT signalling pathway, furthermore, the inhibitor of PI3K or the selective inhibitor of AKR1C2 enzyme activity could reverse the aggressiveness and showed synergistic antitumour effect when combined with cisplatin, both in vitro and in vivo. In conclusion, Our findings revealed that AKR1C2 could function as an oncogene by activating the PI3K/AKT pathway, as a novel prognostic biomarker and/or as a potential therapeutic target to ESCC.

## 1 INTRODUCTION

Oesophageal carcinoma (EC) is the ninth most common aggressive malignancy worldwide and is ranked as the sixth leading cause of cancer‐related mortality.^1^ China has a high incidence of EC, accounting for more than 50% of its global morbidity and mortality. More than 90% of EC patients in China are classified as oesophageal squamous cell carcinoma (ESCC).^2^ The outcome of EC is poor, with a dismal 5‐year overall survival (OS) rate at ~20%‐30%,^3^, ^4^ due to its poorly understood the underlying pathological molecular mechanisms.^3^, ^5^, ^6^

At present, many medical scientists are trying to find effective targets for the treatment of ESCC, but the targeted therapy trials have thus far been disappointing. EGFR is one of the most investigated molecular targets in the field of EC, but the results of clinical studies did not yield a significant survival benefit for EC, including ESCC.^7^, ^8^, ^9^ Therefore, there is no effective targeted drug for the treatment of EC so far, and effective biomarkers selection is urgently warranted. In addition, platinum‐based chemotherapy regimens are the main chemotherapy regimens for EC; however, it poorly responses to chemotherapy,^10^ and the underlying mechanism is unclear.

Aldo‐keto reductases family 1 member C2 (AKR1C2), a member of the aldo/keto reductase superfamily, can metabolize dihydrotestosterone (DHT) into 5α‐androstane‐3α,17β‐diol (3α‐diol).^11^, ^12^ It has been discovered pivotal in the development of urogenital,^11^, ^13^, ^14^ gastrointestinal ^15^, ^16^, ^17^, ^18^, ^19^, ^20^, ^21^, ^22^ cancers and more.^23^, ^24^, ^25^, ^26^, ^27^ However, previous findings on the exact significance of AKR1C2 have been inconsistent and highly debatable, we even don't know whether to regard it as a promoter or suppresser of cancers, and how it participates in the pathogenesis and development of ESCC have not been fully investigated.

In the present study, we demonstrated that AKR1C2 was highly expressed in ESCC patients' tissues and ESCC cell lines. Its high expression was associated with poor prognosis of ESCC patients. Our findings further indicated that AKR1C2 could act as an oncogene, was associated with cisplatin resistance and might be a potential therapeutic target for ESCC treatment.

## 2 MATERIALS AND METHODS

### 2.1 Clinical samples

Pathologically confirmed ESCC paraffin‐tissues were obtained from the Department of Pathology in Sun Yat‐Sen University Cancer Center (SYSUCC) and the First Affiliated Hospital of Sun Yat‐Sen University (FAHSYSU), named the training cohort and the validation cohort, respectively. Another 43 pairs of fresh primary ESCC and the corresponding normal tissues were collected from the Department of Thoracic Surgery in FAHSYSU and stored at liquid nitrogen until use. Written informed consent was obtained from all patients, the study was approved by the Medical Ethical Committee of the SYSUCC and the FAHSYSU, performed in compliance with the Helsinki Declaration.

### 2.2 Cell lines and culture

Cells used in the present study were one immortalized normal oesophageal cell line (NE1) and seven ESCC cell lines (KYSE30, KYSE180, KYSE410, KYSE510, KYSE520, EC18, EC109), which were kindly gifted by professor Guan (Department of Clinical Oncology, the University of Hong Kong). All cell lines were free of mycoplasma infection. They were cultured in DMEM (Gibco) supplemented with 10% FBS (Gibco) and 1% penicillin streptomycin mixed solution in 5% CO_2_ at 37°C.

### 2.3 Real‐time PCR

Total RNA was extracted with TRIzol (Invitrogen) following the manufacturer's instructions. Reverse transcription was performed using the PrimeScript RT Master Mix Kit (TAKARA). RT‐qPCR was carried out using SYBR Green Master Mix (YEASEN), followed by Roche 96/384 holes Real‐Time PCR system (Roche). GAPDH was used as an internal control. The primers used are listed in Table S1.

### 2.4 Western blotting

Western blotting was performed according to previously described standard methods.^28^ Antibodies were used to against AKR1C2 (Abcam), phospho‐AKT Ser473 (Affinity), total AKT (Proteintech), cleaved‐PARP(Affinity), caspase3 (Cell Signaling Technology), E‐cadherin (Proteintech), N‐cadherin (Proteintech), Vimentin (Proteintech), Androgen receptor (AR) (Invitrogen), GAPDH (Proteintech). Dilution ratio used was according to the manufacturer's instructions. GAPDH was used as an internal reference.

### 2.5 Immunohistochemical (IHC) staining

Immunohistochemical was performed according to previously described standard method.^29^ Antibodies were used to against AKR1C2 (Abcam), pAKT S473 (Affinity), Ki‐67 (Cell Signaling Technology), cleaved‐caspase 3 (SAB). IHC staining was reviewed and scored separately by two independent pathologists who were blinded to the patients' characteristics and scoring discrepancies were resolved by mutual consensus. The scoring procedures and method were similar to previously described.^29^ The median scores of AKR1C2 (score = 8) were used as the cut‐off value to classify the patients into a high expression (score ≥ 8) or low‐expression (score < 8) group.

### 2.6 Small interfering RNA (siRNA) and plasmid transfection

SiRNA targeting AKR1C2 and scramble negative control were designed and synthesized by GenePharm. ESCC cells were transfected with the siRNA using the Oligofectamine transfection reagent RNAiMAX (Invitrogen) according to the manufacturer's instructions. The overexpression plasmid and empty vector plasmid were purchased from Genechem and were transfected into the ESCC cells using the X‐tremeGENE™ HP DNA Transfection Reagent (Roche) based on the manufacturer's instructions. After 72 hours, the transfection efficacy was evaluated by RT‐qPCR and Western blotting. Information regarding the siRNAs and plasmids of AKR1C2 are listed in Tables S3 and S4.

### 2.7 Lentiviral‐mediated knockdown assay

For AKR1C2 knockdown assays, one lentiviral constructs containing short hairpin RNAs (shRNA) specifically targeting AKR1C2 were purchased from GeneCopoeia and were packaged in the 293T cells. Virus‐containing supernatants were collected and stably transfected into KYSE180 and EC109 cells. Empty vector transfected cells were used as controls and selected stably transduced cells by 2.5 mg/mL puromycin (Sigma) for 7 days. The sequences of shRNA against AKR1C2 is listed in Table S3.

### 2.8 Function studies and drug treatment experiments

Protocols for cell proliferation, migration assays and drug treatment assays are provided in Appendix S1.

### 2.9 Animal experiment

The experiments with mice were approved by the Research Animal Resource Center of Sun Yat‐Sen University (Approval number: L102042019060D). Protocols for establishing human ESCC xenografts and drug treatment assays are provided in Appendix S1.

### 2.10 Statistical analysis

Statistical analyses were performed using GraphPad Prism 8, SPSS version 21.0 (IBM). The relationship between AKR1C2 expression and clinicopathological characteristics were analysed using the Pearson chi‐square test. Kaplan‐Meier analysis with log‐rank test was applied for survival analysis. Univariate and multivariate Cox proportional hazard regression models were used to evaluate the survival hazard with a forward stepwise procedure. For functional assays analysis, when the data were normally distributed, the comparison between two groups was conducted using the Student's *t* test. *P* < .05 was considered as statistically significant.

## 3 RESULTS

### 3.1 AKR1C2 expression was distinctively up‐regulated in ESCC

**FIGURE 1:**
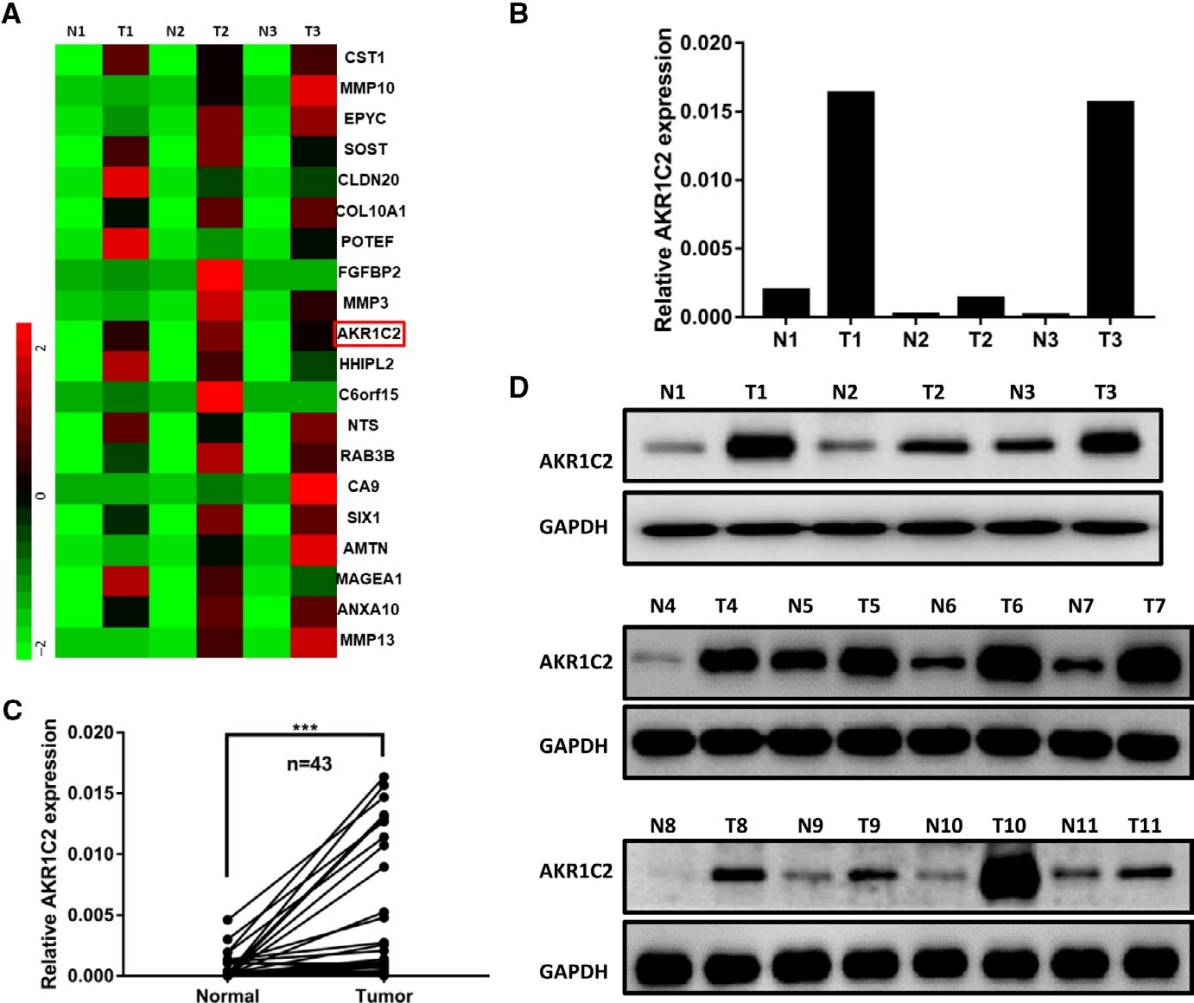
AKR1C2 was significantly up‐regulated in ESCC. A, The top 20 up‐regulated genes in three pairs of matched adjacent normal/tumour samples. B, Validation of altered AKR1C2 expression in three pairs tumour and corresponding adjacent normal samples by RT‐qPCR. C, AKR1C2 mRNA level was detected by RT‐qPCR in 43 pairs ESCC and matched normal tissues. ***P < .001. Student *t* test. D, Protein level of AKR1C2 was determined in 11 pairs of ESCC and matched normal tissues, including the three pairs of RNA‐sequencing samples

The expression of the top 20 up‐regulated genes was shown in Figure 1A, among which AKR1C2 was the prominent candidate gene of interest for the following reasons: first, it was significantly up‐regulated in the three pairs of RNA‐sequencing samples; second, its reported functions were controversial, even in the same kind of tumour; third, there were few studies about AKR1C2 in EC, including ESCC and oesophageal adenocarcinoma. Then, RT‐qPCR (Figure 1B) was performed to validate the result of RNA‐sequencing. To investigate whether the up‐regulation of AKR1C2 was a common event in ESCC, the mRNA expression of AKR1C2 in 43 paired samples were detected by RT‐qPCR and was found to be elevated in most of ESCC tissues compared to the corresponding adjacent normal tissues (*P* < .001; Figure 1C). Consistently, the protein expression of AKR1C2 was elevated in 11 paired samples, including the three pairs of RNA‐sequencing samples (Figure 1D). In addition, data from TCGA (Figure S1A) and Oncomine databases (Figure S1B,C) further confirmed that AKR1C2 was elevated in ESCC.

### 3.2 AKR1C2 was associated with advanced clinicopathological features and worse survival as an independent prognosis factor

**FIGURE 2:**
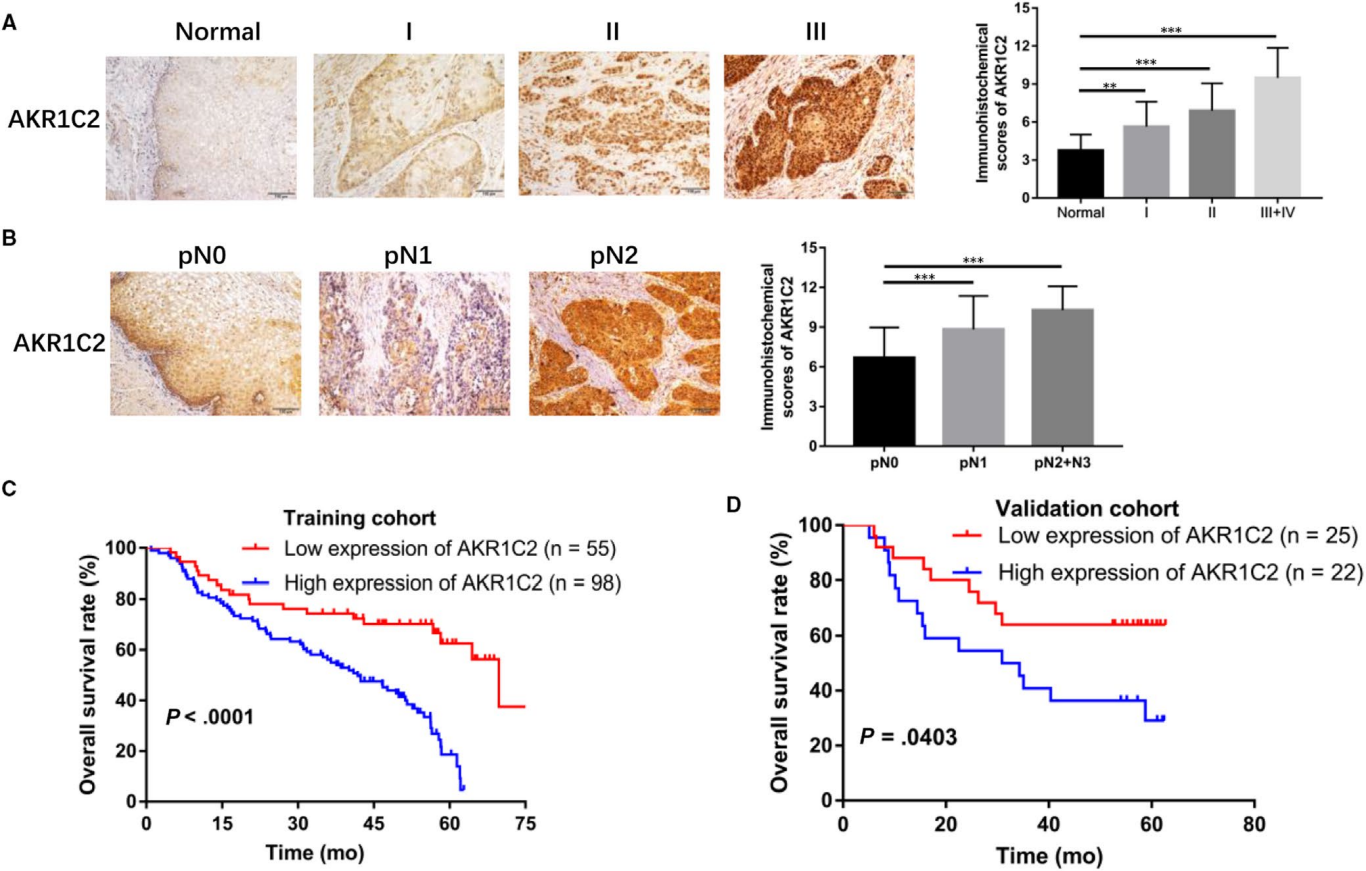
High expression of AKR1C2 was identified in ESCC tissues and was associated with advanced clinicopathological features and worse survival. A, B, AKR1C2 expression in 153 ESCC and 20 adjacent normal paraffin‐embedding tissues were detected by IHC, representative images of different pathological stage (A) and lymph node metastasis (B). Pictures taken at 200× original magnification. Scale bar, 100 μm. **P < .01, ***P < .001. Student *t* test. C, D, Kaplan‐Meier analysis revealed that patients with high levels of AKR1C2 expression had reduced survival times compared to patients with low levels, in both training cohort and validation cohort

To further investigate the frequency of AKR1C2 up‐regulation in ESCC, IHC was performed in 200 paraffin‐embedded ESCC tissues (153 cases from the training cohort and 47 cases from the validation cohort) and 20 adjacent normal oesophageal tissues. The adjacent normal oesophageal tissues were found to have low or undetectable level of AKR1C2 but was highly expressed in the ESCC tissues (98/153 in training cohort and 34/47 in validation cohort). Furthermore, AKR1C2 expression was gradually increased from stage I to stage Ⅳ (Figure 2A). Consistently, AKR1C2 expression was found to gradual increase expression from pN0 to pN3 in ESCC patients (Figure 2B).

According to the cut‐off value (median IHC score = 8) of AKR1C2 staining, patients were classified into a low (score < 8) or high (score ≥ 8) AKR1C2 expression group. Next, we explored the correlation between AKR1C2 expression and the clinicopathological parameters of patients with ESCC. Chi‐square test was performed to evaluate the clinicopathological features between the two groups in the training cohort. As shown in Table 1, strong associations were observed between AKR1C2 expression to pT stage (*P* = .005), pN stage (*P* = .000), pathological stage (*P* = .000) and vascular invasion (*P* = .003). The characteristics of the patients in the validation cohorts were attached in Table S2.

**TABLE 1:** Correlation of AKR1C2 and clinicopathological parameters in the training cohort

Variables	AKR1C2 expression				
	Cases (%)	Low expression	High expression	χ^2^	*P* value
Age^a^					
<61	70 (45.8)	30	40	2.675	.128
≥61	83 (54.2)	25	58		
Gender					
Male	122 (79.7)	42	80	0.605	.473
Female	31 (20.3)	13	18		
BMI					
<18.5	19 (12.4)	8	11	1.731	.421
18.5‐23.9	102 (66.7)	33	69		
≥24	32 (20.9)	14	18		
Smoking status					
Yes	102 (66.7)	35	67	0.355	.551
No	51 (33.3)	20	31		
Alcohol consumption					
Yes	71 (46.4)	25	46	0.031	.860
No	82 (53.6)	30	52		
Family history					
Yes	16 (10.5)	6	10	0.019	.891
No	137 (89.5)	49	88		
Tumour location					
Upper	16 (10.5)	3	13	2.857	.240
Middle	95 (62.0)	38	57		
Lower	42 (27.5)	14	28		
Differentiation					
Well	24 (15.7)	7	17	0.688	.709
Moderate	91 (59.5)	33	58		
Poor	38 (24.8)	15	23		
pT status					
T1	9 (5.9)	7	2	12.677	.005
T2	26 (17.0)	13	13		
T3	114 (74.5)	35	79		
T4	4 (2.6)	0	4		
pN status					
N0	74 (48.4)	45	29	41.022	.000
N1	54 (35.3)	10	44		
N2	19 (12.4)	0	19		
N3	6 (3.9)	0	6		
Pathological stage					
Ⅰ	19 (12.4)	15	4	54.238	.000
Ⅱ	58 (37.9)	34	24		
Ⅲ	74 (48.4)	6	68		
Ⅳ	2 (1.3)	0	2		
Vascular invasion					
Yes	30 (19.6)	4	26	8.288	.003
No	123 (80.4)	51	72		
Nerve tract invasion					
Yes	40 (26.1)	10	30	2.819	.093
No	113 (73.9)	45	68		

^a^ Median age.

To explore the association between AKR1C2 expression and the prognosis of ESCC patients, we evaluated the correlation between AKR1C2 expression and clinical outcomes. For the training cohort, the median OS in the high and low AKR1C2 expression groups were 41.8 and 69.7 months, respectively. As shown in Figure 2C, a high expression of AKR1C2 was correlated to a poorer prognosis in patients with ESCC (*P* < .0001) and was further confirmed in the validation cohort (*P* = .04; Figure 2D). Univariate and multivariate analyses (Cox proportional hazards regression model) performed in the training cohort showed that age, BMI, pathological stage, vascular invasion, as well as AKR1C2 expression were independent prognostic factors for OS (Table 2).

**TABLE 2:** Univariate and multivariate analyses of clinicopathological parameters and AKR1C2 for overall survival in the training cohort

Variables	Univariate analysis		Multivariate analysis	
	HR (95% CI)	*P* value	HR (95% CI)	*P* value
Age	1.567 (1.024‐2.397)	.038	1.625 (1.047‐2.523)	.031
Gender	1.451 (0.833‐2.527)	.189		
BMI	0.574 (0.397‐0.830)	.003	0.546 (0.376‐0.794)	.002
Smoking status	1.056 (0.683‐1.632)	.806		
Alcohol consumption	1.210 (0.798‐1.835)	.369		
Family history	0.480 (0.194‐1.187)	.112		
Tumour location	1.167 (0.826‐1.649)	.381		
Differentiation	1.166 (0.835‐1.630)	.368		
pT status	1.553 (1.071‐2.251)	.020		
pN status	1.800 (1.444‐2.244)	.000		
Pathological stage	1.938 (1.397‐2.690)	.000	1.458(1.008‐2.107)	.045
Vascular invasion	2.189 (1.368‐3.504)	.001	1.866 (1.131‐3.077)	.015
Nerve tract invasion	1.119 (0.696‐1.798)	.643		
AKR1C2 expression	3.092 (1.821‐5.249)	.000	2.126 (1.190‐3.797)	.011

Abbreviations: CI, confident interval; HR, hazard ratio.

### 3.3 AKR1C2 promotes ESCC cells proliferation in vitro and in vivo

**FIGURE 3:**
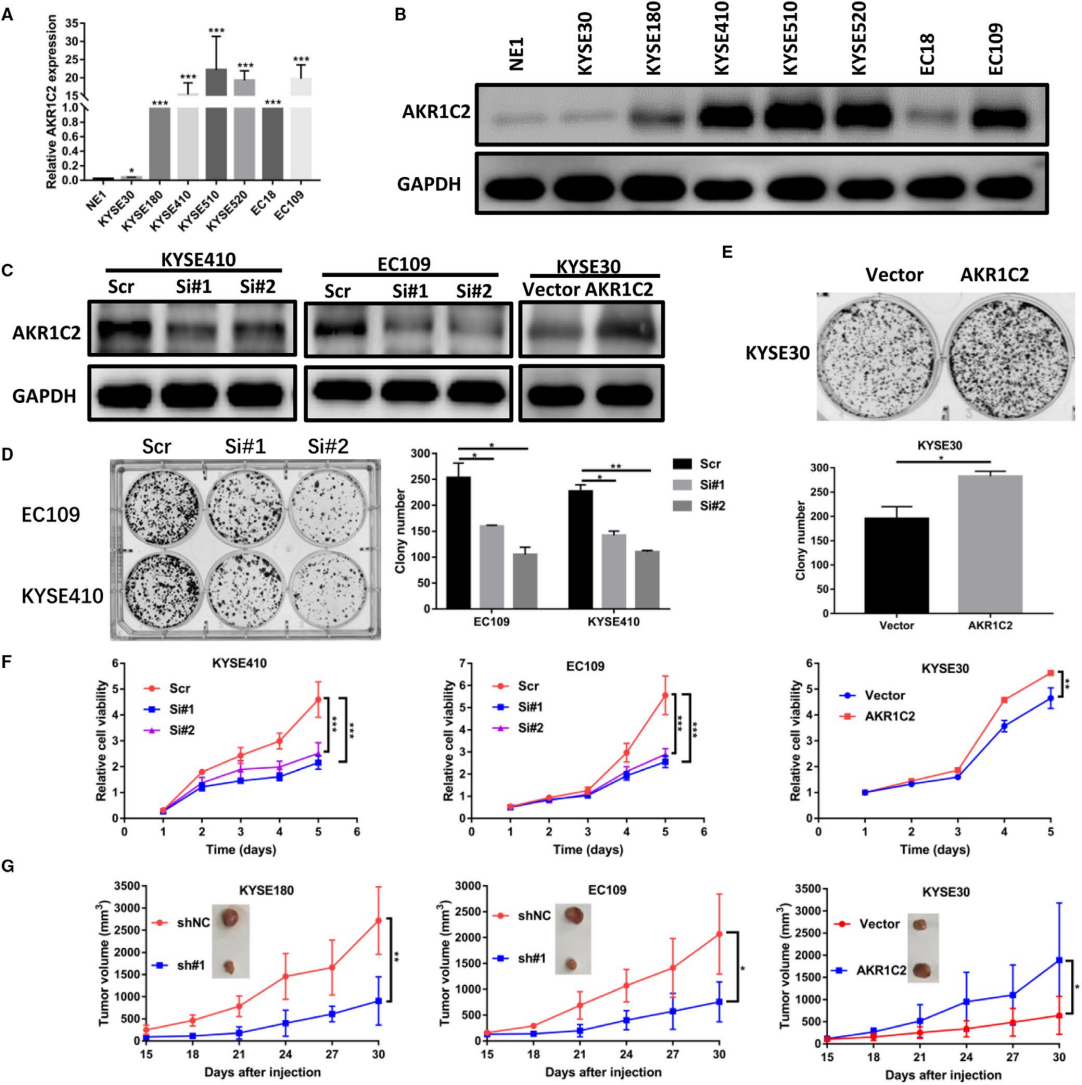
AKR1C2 promotes ESCC cell proliferation in vitro and in vivo. A, B, Analysis of AKR1C2 mRNA and protein levels in ESCC cell lines compared with an immortalized normal oesophageal cell line (NE1) by RT‐qPCR (*P < .05, ***P < .001. Student *t* test.) and Western blotting. C, Decreased or overexpression of AKR1C2 were confirmed by Western blotting after knockdown or overexpression in KYSE410, EC109 and KYSE30 cells. D, Colony formation assays of EC109 and KYSE410 cells transfected with knockdown‐AKR1C2. E, Colony formation assays of KYSE30 cell transfected with an overexpression plasmid. F, MTS assays were performed to determine the cell proliferation of KYSE410, EC109 cells after transfection of siRNA and KYSE30 cell after transfection of overexpression plasmid. G, ESCC cells after gene manipulation and corresponding control cells were subcutaneously injected into the right flank of nude mice for 30 d. The average of tumour size was measured. Data were presented as the mean ± SD. *P < .05, **P < .01, ***P < .001. Student *t* test

To explore the role of AKR1C2 in ESCC, we identified the AKR1C2 expression level in ESCC cell lines. As shown in Figure 3A,B, both the mRNA and protein levels of AKR1C2 in ESCC cell lines were significantly higher than the immortalized normal oesophageal cell line (NE1). Based on the loss and gain of function, the function of AKR1C2 in the ESCC cell lines was further investigated. According to the results of RT‐qPCR and Western blotting, we defined KYSE30 as AKR1C2 relatively low‐expression cell line and others ESCC cells were AKR1C2 relatively high‐expression cell lines. Therefore, we transfected KYSE410 and EC109 cells with AKR1C2 siRNA or scramble siRNA, transfected KYSE30 with overexpression plasmid or empty vector plasmid, and the manipulations were confirmed by RT‐qPCR (Figure S2A‐C) and Western blotting (Figure 3C).

Then, colony formation assays were performed and showed that the knockdown of AKR1C2 significantly suppressed the proliferation of KYSE410 and EC109 cells as compared to the control groups (Figure 3D). By contrast, the overexpression of AKR1C2 promoted the proliferation of KYSE30 cell (Figure 3E). Results from MTS assays further confirmed these findings (Figure 3F). To further investigate whether AKR1C2 affects cell proliferation in vivo, the shAKR1C2 mediated knockdown in KYSE180 and EC109 cells and overexpressed plasmid mediated in KYSE30 cell were inoculated into BALB/C nude mice. As shown in Figure 3G, the knockdown of AKR1C2 could significantly inhibit the in vivo tumour growth while overexpressed AKR1C2 could promote them. These results were further confirmed by IHC experiments (Figure 5E).

### 3.4 AKR1C2 promotes ESCC cells migration by inducing epithelial‐mesenchymal transition (EMT)

**FIGURE 4:**
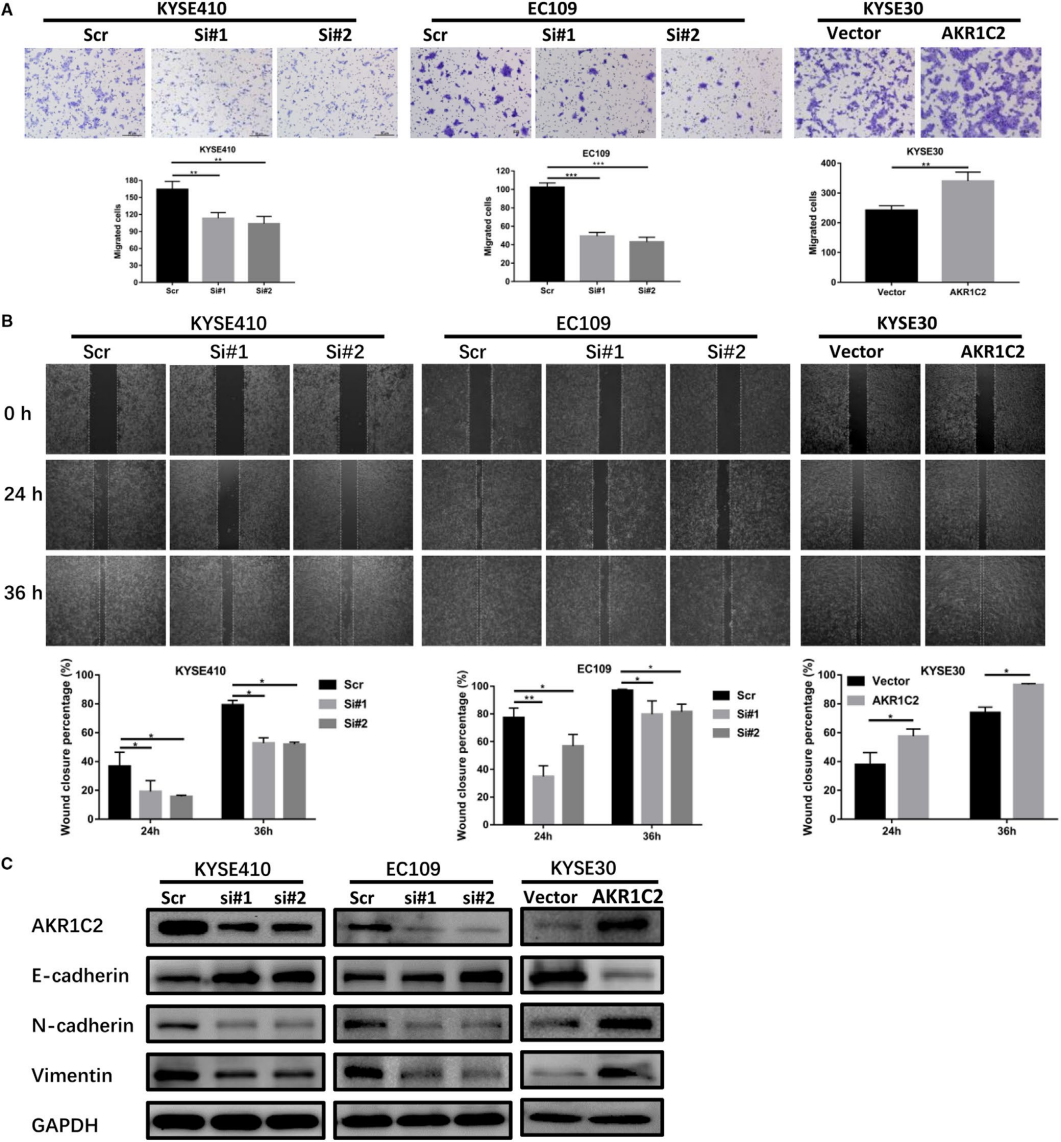
AKR1C2 promotes ESCC cells migration by inducing EMT. A, Migration assays were used to investigate the change of migratory ability of KYSE410, EC109 and KYSE30 cells after transfection, respectively. **P < .01, *** P < .001. Student *t* test. B, Wound‐healing assays were conducted to detect change in migratory ability after silencing or overexpression of AKR1C2 in KYSE410, EC109 and KYSE30 cells. *P < .05. Student *t* test. C, EMT markers were detected by Western blotting in the indicated cells

To explore the biological function of AKR1C2 in ESCC, migration assays were performed in KYSE410 and EC109 cells which were knockdown by siRNA. The results revealed that the knockdown of AKR1C2 significantly inhibited cells migration as compared to scramble control groups. However, ectopic overexpression of AKR1C2 in KYSE30 cell had the opposite effect (Figure 4A). Wound‐healing assays confirmed the effect of AKR1C2 on cell migration as it was found that cells motility were repressed when AKR1C2 was silenced but promoted when AKR1C2 was overexpressed (Figure 4B). The EMT markers were detected by Western blotting to investigate the mechanism of migration mediated by AKR1C2. As shown in the Figure 4C, compared with the control groups, the epithelial markers (E‐cadherin) were increased and the mesenchymal markers (N‐cadherin, vimentin) were reduced in the knockdown groups. Conversely, AKR1C2 overexpression displayed a reversed trend in the expression of EMT markers. To further validated the effect of AKR1C2 on cell metastasis in vivo, KYSE180 and EC109 cells stably transfected with shAKR1C2 or control vector were injected into the tail veins of nude mice, and the mice were euthanized 3 months after injection. The lungs were weighted, then stained with haematoxylin and eosin (HE), the lung metastasis numbers were counted under naked eye and high magnification microscope fields, but no metastatic lesions were found.

### 3.5 PI3K/AKT signalling pathway is regulated by AKR1C2

**FIGURE 5:**
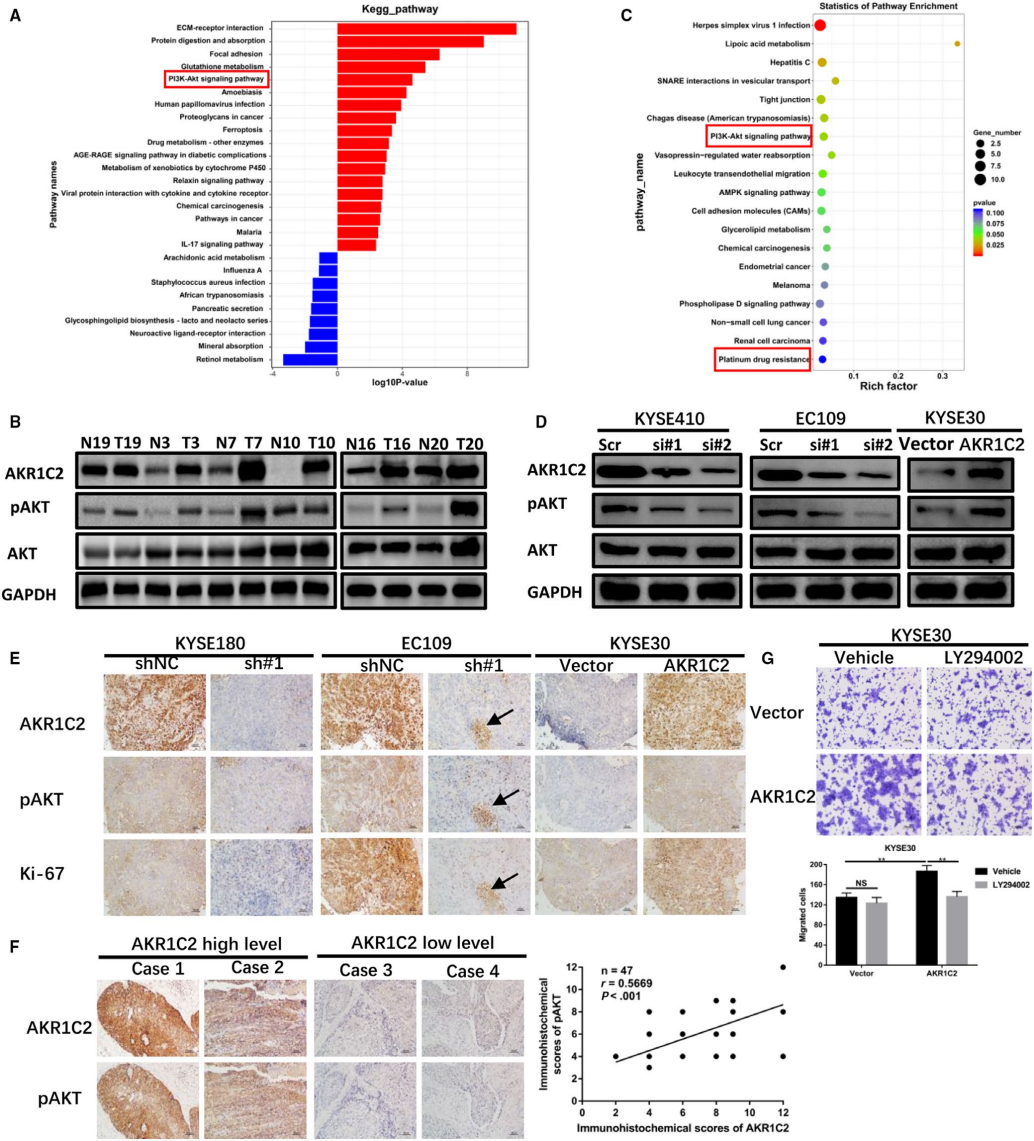
PI3K/AKT signalling pathway was regulated by AKR1C2. A, Three pairs of matched tumour/normal tissues were performed with RNA‐sequencing and the KEGG results were shown, and the PI3K/AKT signalling pathway was circled with a rectangle. B, The pAKT protein level was detected by Western blotting in six pairs of ESCC tissues and corresponding adjacent normal tissues. C, The KYSE30 cell was transfected with an empty vector or AKR1C2 plasmid for 72 h, mRNA sequencing analysis was performed, and the KEGG results were shown, the PI3K/AKT signalling pathway and platinum drug resistance were highlighted by rectangles. D, Western blotting analysis the change of pAKT in the indicated ESCC cell lines. E, The IHC was performed to detect the AKR1C2‐mediated expression change of pAKT and Ki‐67 in the excised tumours from tumour growth models. The arrows indicated regional areas, suggesting a positive correlation between the AKR1C2, pAKT and Ki‐67. The pictures were taken at 200× original magnification. Scale bar, 50 μm. F, The correlation between AKR1C2 and pAKT expression was analysed by IHC in 47 ESCC clinical samples (*r* = .5669, *P* < .001). Representative images of AKR1C2 high‐expression and low‐expression cases, 200× original magnification. Scale bar, 50 μm. G, The transfected KYSE30 cell were treated with vehicle (DMSO) or PI3K inhibitor LY294002 (10 μmol/L) for 24 h, change in migratory ability was detected with Migration assays. **P < .01, NS, not significant. Student *t* test

The results of RNA‐sequencing followed by KEGG analysis of three pairs matched tissues indicated that PI3K/AKT signalling pathway was involved in the ESCC tumorigenesis (Figure 5A) and was validated by six pairs of matched tissues by Western blotting (Figure 5B). To further screen the signalling pathway affected by the AKR1C2 in ESCC, KYSE30 cell transfected with AKR1C2 plasmid or empty control plasmid were performed with RNA‐sequencing. Next, the KEGG pathways were analysed and the canonical PI3K/AKT signalling pathway signalling was found to be significantly enriched (Figure 5C). Moreover, the results of Western blotting confirmed that, compared with the control groups, phosphorylated AKT (pAKT) was reduced in the knockdown cells and increased in the overexpressed cell (Figure 5D). This was further confirmed in ex vivo xenograft tumour samples by IHC which exhibited positive correlation with Ki‐67 (Figure 5E). Consistently, AKR1C2 expression also showed positive correction with pAKT in the paraffin‐embed ESCC tissues (n = 47, *r* = .5669, *P* < .001; Figure 5F). Furthermore, LY294002, an inhibitor of PI3K, was applied to detect the effect of PI3K/AKT signalling pathway on AKR1C2‐mediated cell migration by migration assays. As shown in Figure 5G, LY294002 could significantly attenuate cell migration. Moreover, the pAKT level could be restrained by LY294002 accompanied with the increased E‐cadherin and reduced Vimentin in the AKR1C2 overexpression cell. (Figure S3).

### 3.6 AKR1C2 overexpression resulted in cisplatin resistance in ESCC

**FIGURE 6:**
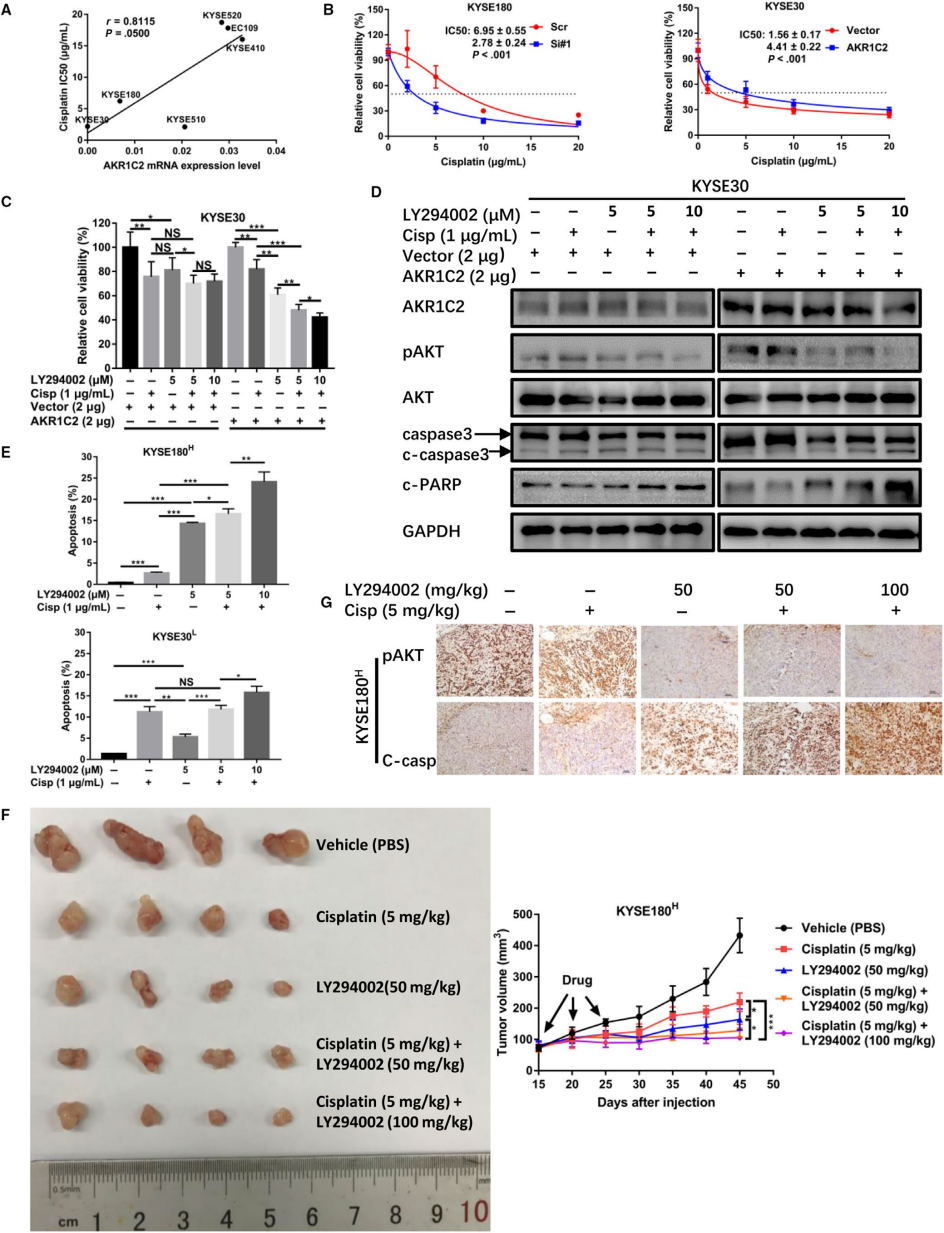
AKR1C2 overexpression resulted in cisplatin resistance in ESCC. A, The mRNA level of AKR1C2 in six ESCC cell lines showed a significantly positive correlation with the IC50 of cisplatin (*r* = .8115, *P* = .05). B, AKR1C2 expression level significantly affected the IC50 of cisplatin in AKR1C2 knockdown cell (left) and overexpression cell (right). C, MTS assays were performed to detected cells viability after cisplatin, LY294002 single or combinational use in indicated cells for 72 h. D, Indicated cells were treated with cisplatin and LY294002 at different combination scheme for 72 h, the pAKT, cleaved‐caspase3, cleaved‐PARP protein levels were analysed by Western blotting. GAPDH was used as the loading control. E, After drug exposure for 72 h, KYSE30^L^ and KYSE180^H^ were double stained with propidium iodide (PI) and Annexin V‐FITC and analysed by flow cytometry. F, Tumour volumes of KYSE180^H^ xenografts treated with different combination scheme by intraperitoneal injection every 5 d and for three cycles. Vehicle, PBS. G, Representative images of pAKT and c‐aspase3 IHC staining of xenograft tumours. 200× original magnification. Scale bar, 50 μm. Cisp, cisplatin; C‐casp, cleaved‐caspase3. Superscript “L” and “H” represent low expression and high expression, respectively. *P < .05, **P < .01, ***P < .001, NS, not significant. Student *t* test

Platinum drugs are the basic drugs used as chemotherapy for locally advanced or advanced EC but it has been found that most of the patients had decreased sensitivity to cisplatin. To study whether AKR1C2 was involved in the reported platinum resistance, the IC50 of cisplatin was determined in six ESCC cell lines. Our findings showed that the cisplatin IC50 was positively correlated with AKR1C2 expression in ESCC cell lines (*r* = .8115, *P* = .05; Figure 6A). Next, we found that overexpressing AKR1C2 attenuated the sensitivity to cisplatin while knocking it down had a reversed effect (Figure 6B). KEGG pathway analysis also showed that AKR1C2 had potential involvement in the platinum drug resistance (Figure 5B).

Based on our above findings that AKR1C2 mediated cisplatin resistance and activated the PI3K/AKT signalling pathway, we attempted to combine cisplatin and LY294002 to treat indicated cells to explore whether this combination therapy would have a synergistic antitumour effect. After drugs were added to the indicated cells for 72 hours, cell viability was tested by MTS. As shown in Figure 6C and S4A, the combinational use of cisplatin plus LY294002 had a more obvious inhibition effect in the AKR1C2 overexpression cell as compared to the vector control group and showed the dose‐dependent effect of LY294002. Furthermore, Western blotting was performed to examine the levels of pAKT and apoptosis markers (cleaved‐caspase3 and cleaved‐PARP). As shown in Figure 6D, in the combination treatment groups, pAKT was inhibited and accompanied with an increase in apoptosis markers compared to the single drug groups. This phenomenon was more distinctive in the AKR1C2 overexpression cell and also showed the LY294002 dose‐dependent effect.

Next, to validate the above findings, we chose two parental ESCC cell lines, namely KYSE30^L^ and KYSE180^H^, to which the phosphoinositide (PI)/Annexin V‐FITC apoptosis assays were performed. As shown in Figures S4B and 6E, KYSE180^H^ had greater apoptosis ratio in the combination treatment groups compared to KYSE30^L^.

Then, a KYSE180^H^ xenograft model was established in nude mice to investigate the in vivo effects of the above findings. The results showed that the tumour volume was much smaller in the combination therapy groups as compared to those treated with the single drug (cisplatin or LY294002) groups (Figure 6F). Additionally, the results were further evaluated by IHC staining of c‐caspase3 in ex vivo tumour samples (Figure 6G).

### 3.7 AKR1C2 can be a potential therapeutic target

**FIGURE 7:**
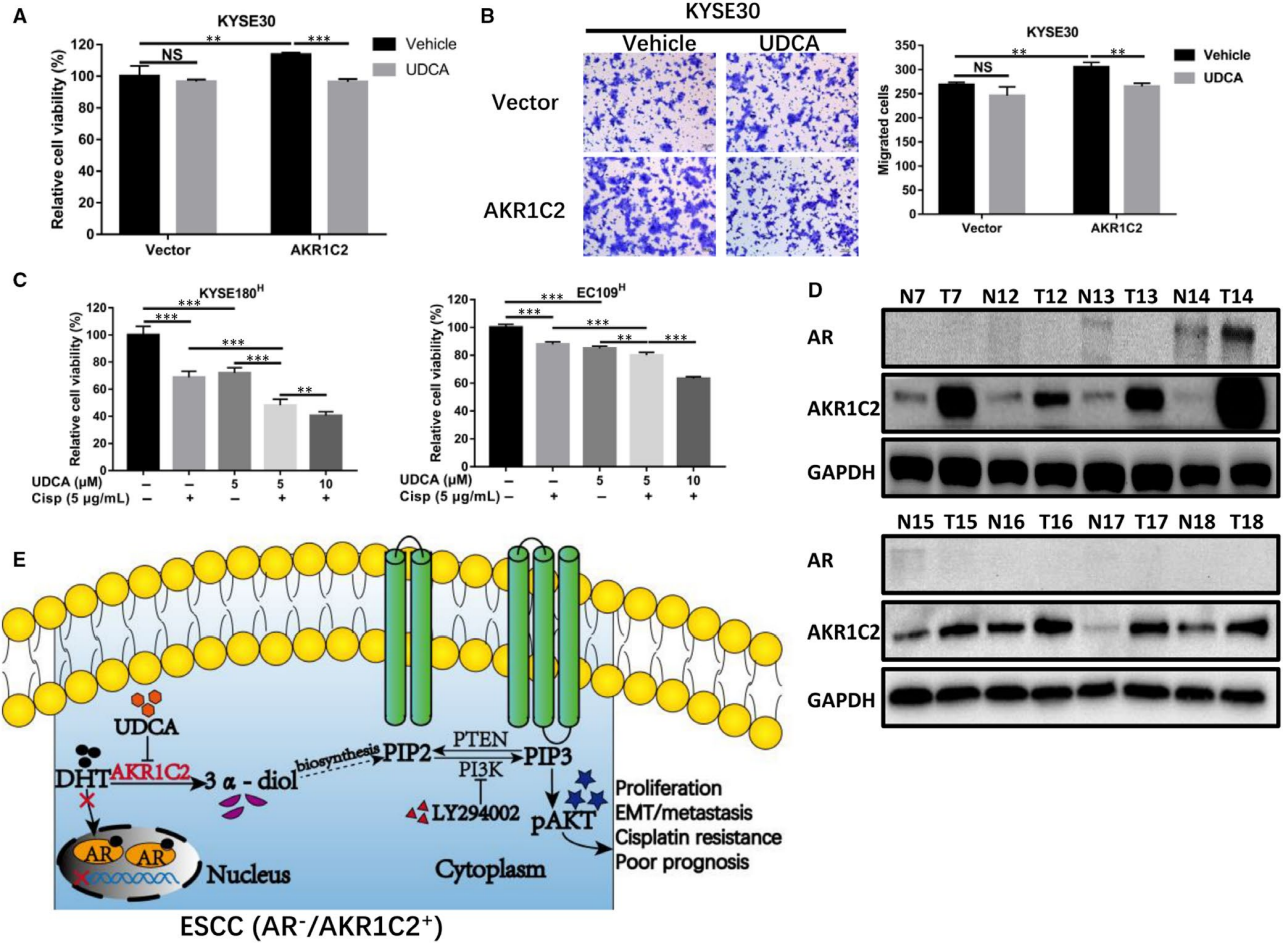
AKR1C2 can act as a potential therapeutic target. A, The cell viability was detected by MTS assays after UDCA was added to the indicated cells for 24 h. B, After treatment with UDCA for 24 h, Migration assays were performed to estimate the change in migratory ability compared with the control group. **P < .01, NS, not significant. Student *t* test. C, MTS assays were performed to detect cell viability after cisplatin, UDCA single or combination therapy for 72 h in KYSE180^H^ and EC109^H^ cells. D, AR protein level was detected in eight pairs of ESCC and corresponding normal tissues by Western blotting. E, Proposed model illustrating the tumorigenesis effect of AKR1C2 in the pathogenesis of ESCC. Vehicle, absolute ethanol. Superscript "H" represent high expression. **P < .01, ***P < .001, NS, not significant. Student *t* test

KYSE30 cell transfected with AKR1C2 plasmid or empty control plasmid was performed with RNA‐sequencing, following GO analysis results inferred that AKR1C2 overexpression accompanied with the functional changes of inositol metabolism (Figure S5A), and the KEGG pathway analysis of EC109 cell suggested that the steroid and steroid hormone biosynthesis maybe change when AKR1C2 was knocked down (Figure S5B). Ursodeoxycholic acid (UDCA), a selective inhibitor of AKR1C2 enzymatic activity, was used to treat indicated cells followed by MTS (Figure 7A) and migration assays (Figure 7B). The results indicated that UDCA could inhibit the proliferation and migration in AKR1C2‐overexpressed cells but not the control group. Further, the AKR1C2 inducing EMT was found to be reversed by UDCA (Figure S5C). Next, KYSE180^H^ and EC109^H^ were chosen for treating with cisplatin, UDCA alone or combination therapy. The results showed that UDCA could repress the growth of high AKR1C2 expression ESCC cells and had a synergistic killing effect on ESCC cells even in an UDCA dose‐escalation manner when combined with cisplatin (Figure 7C).

AKR1C2 is the metabolic enzyme of steroid hormone DHT, which could recognize and bind with AR to activate the DHT‐AR pathway. Therefore, we explored the AR protein level in ESCC clinical samples. Our results showed that that AR was barely expressed in the eight pairs of matched ESCC tissues (Figure 7D).

In light of the findings observed in this present study, a proposed model illustrating the oncogenic effect of AKR1C2 in the pathogenesis of ESCC is provided in Figure 7E.

## 4 DISCUSSION

There have been debates regarding the role of AKR1C2 in tumours. For instance, Wang et al^14^ and Huang et al^22^ reported that AKR1C2 functioned as an oncogene in human prostate cancer whereas Ji et al^11^ obtained opposing results, supporting that AKR1C2 acted as a tumour suppressor gene in human prostate cancer. Similarly, its specific role in the progression of ESCC is also still far from being fully elucidated.

In the present study, functional assays revealed that the overexpression of AKR1C2 promoted cells proliferation and migration in vitro and in vivo. Conversely, silencing AKR1C2 exhibited opposing effects. Several prior studies have also demonstrated similar findings regarding AKR1C2 in different cancers.^20^, ^21^ Nevertheless, we did not find metastatic nodules in lung metastasis models. A reasonable explanation for this might be that ESCC metastasizes mainly through lymph nodes and less often through blood route and that was the reason why ESCC patients were less likely to have lung metastases, consistent observations in clinical practice. These results suggest that AKR1C2 may act as an oncogene in ESCC and may be a potential therapeutic target.

In additional, the PI3K/AKT pathway has been described as a major pathway frequently activated in human ESCC.^30^, ^31^ However, except one previous study which reported that a high expression of AKR1C2 could provoke the PI3K/AKT signalling pathway in prostatic cancer,^14^ studies concerning signal pathways mediated by AKR1C2 are limited, and the function of AKR1C2 and its modulation of PI3K/AKT signalling pathway has not been investigated in ESCC. Here, we discovered that AKR1C2 mediated the PI3K/AKT pathway activation in ESCC and regulated cell proliferation, migration and cisplatin resistance. The AKR1C2 mRNA level had a positive correlation with the IC50 of cisplatin in ESCC cell lines. Thus, a high expression of AKR1C2 in ESCC is likely to confer to resistance to cisplatin use, making it a prime candidate for combination with other safe reagents for cancer treatment. In addition, the AKR1C2 mediated PI3K/AKT signalling pathway was activated, co‐occurrence of alterations suggested combination therapy opportunity.^32^ Interestingly, cisplatin plus LY294002 combination therapy demonstrated significant improvement of antitumour effect in vitro and in vivo, in a LY294002 dose‐dependent manner, especially in the AKR1C2 overexpression cells. Partly consist with our findings, several studied have also found that AKR1C2 regulated cisplatin resistance in other cancers^22^, ^27^, ^33^ but the resistance mechanism was sti eously injected into the right flank of nude mice for 30 days. The average of tumour size wll unclear. Besides, several previous studies in other drugs have reported that combined therapy had synergistic antitumour effect.^34^, ^35^, ^36^ Collectively, AKR1C2 promoted cisplatin resistance and cisplatin combine with LY294002 had a synergistic antitumour effect. These findings may provide a promising new guidance for personalized precision therapy in ESCC treatment and hopefully can have potential application in other cancers as well but their corresponding underlying mechanism still warrant further investigation.

The human AKR1C2 can interconvert steroidal hormones from their active to inactive forms,^37^ thus, representing the potential drug target for the development of reagents in the treatment of hormone‐dependent cancers like prostate, breast and endometrial cancers, as well as in other diseases. Our study demonstrated that UDCA, a selective inhibitor of AKR1C2 enzyme activity,^38^ could attenuate the proliferation and migration of ESCC cells and that the combined use of cisplatin with UDCA could increase antitumour effect as compared with single drug. This result is partly in concord with the findings in other cancer.^27^, ^39^ Taken together, these results demonstrated that UDCA could reverse the aggressiveness mediated by AKR1C2, at least in vitro and may be a potential therapeutic target.

Combining the above results, we speculate that the role of AKR1C2 in tumours depends on AR, which has been reported as a pivotal drug target in breast cancer,^40^, ^41^, ^42^ when acting in an AR‐dependent manner, it inhibits the DHT‐AR signalling pathway by metabolizing DHT ^11^, ^23^; On the contrary, when acting in an AR‐independent manner, AKR1C2 metabolizes DHT into 3α‐diol, which has been reported can activate the PI3K/AKT signalling pathway.^43^, ^44^, ^45^, ^46^, ^47^, ^48^, ^49^ In addition, previous studies reported that P) could be chemical synthesized or biosynthesized by diol in vitro.^50^, ^51^ As ESCC tissues hardly expresses AR (Figure 7D), thus, it is reasonable to infer that the metabolites 3α‐diol may be reused to biosynthesis PI, known as a precursor of PI3K,^52^ following by activating the PI3K/AKT pathway in an AR‐independent manner,^43^, ^44^, ^45^, ^46^ However, there is insufficient evidence that whether 3α‐diol could biosynthesis PI directory in vivo. Collectively, these results suggest that for AR negative AKR1C2 positive (AR^−^/AKR1C2^+^) ESCC, AKR1C2 mediates activation of PI3K/AKT pathway may be an alternative to DHT‐AR pathway, which uses androgen metabolites to activate a classic tumour signalling pathway. However, further study is still required to confirm these findings.

## CONFLICT OF INTEREST

The authors confirm that there are no conflicts of interest.

## AUTHOR CONTRIBUTION

**Zhan‐Fei Zhang:** Validation (equal); Writing‐review & editing (equal). **Tie‐Jun Huang:** Validation (equal); Writing‐original draft (equal). **Xin‐Ke Zhang:** Formal analysis (supporting); Methodology (supporting). **Yu‐Jie Xie:** Data curation (equal); Formal analysis (lead); Visualization (equal). **Si‐Ting Lin:** Data curation (equal); Visualization (equal). **Fei‐Fei Luo:** Data curation (equal); Visualization (equal). **Dong‐Fang Meng:** Writing‐review & editing (supporting). **Hao Hu:** Writing‐review & editing (supporting). **Jing Wang:** Conceptualization (equal). **Li‐Xia Peng:** Conceptualization (equal). **Chao‐Nan Qian:** Funding acquisition (supporting). **Chao Cheng:** Supervision (supporting). **BJ Huang:** Funding acquisition (lead); Resources (lead); Supervision (lead).

## Supporting information

Appendix S1

Table S1‐S4

Fig S1‐S5

## Data Availability

The data used to support the findings of the study are available from the corresponding author on reasonable request.

## References

[1] **Bray** F , **Ferlay** J , **Soerjomataram** I , et al. Global cancer statistics 2018: GLOBOCAN estimates of incidence and mortality worldwide for 36 cancers in 185 countries. CA Cancer J Clin. 2018;68:394‐424. 30207593 10.3322/caac.21492

[2] Chen W , Zheng R , Baade PD , et al. Cancer statistics in China, 2015. CA Cancer J Clin. 2016;66:115‐132. 26808342 10.3322/caac.21338

[3] Ohashi S , Miyamoto S , Kikuchi O , et al. Recent advances from basic and clinical studies of esophageal squamous cell carcinoma. Gastroenterology. 2015;149:1700‐1715. 26376349 10.1053/j.gastro.2015.08.054

[4] Zeng H , Chen W , Zheng R , et al. Changing cancer survival in China during 2003–15: a pooled analysis of 17 population‐based cancer registries. Lancet Glob Health. 2018;6:e555‐e567. 29653628 10.1016/S2214-109X(18)30127-X

[5] van den Ende T , Smyth E , Hulshof M , et al. Chemotherapy and novel targeted therapies for operable esophageal and gastroesophageal junctional cancer. Best Pract Res Clin Gastroenterol. 2018;36–37:45‐52. 10.1016/j.bpg.2018.11.005 30551856

[6] Liu Y , Xiong Z , Beasley A , et al. Personalized and targeted therapy of esophageal squamous cell carcinoma: an update. Ann N Y Acad Sci. 2016;1381:66‐73. 27399176 10.1111/nyas.13144 PMC5083215

[7] Burtness B , Goldwasser MA , Flood W , et al. Phase III randomized trial of cisplatin plus placebo compared with cisplatin plus cetuximab in metastatic/recurrent head and neck cancer: an Eastern Cooperative Oncology Group study. J Clin Oncol. 2005;23:8646‐8654. 16314626 10.1200/JCO.2005.02.4646

[8] Crosby T , Hurt CN , Falk S , et al. Chemoradiotherapy with or without cetuximab in patients with oesophageal cancer (SCOPE1): a multicentre, phase 2/3 randomised trial. Lancet Oncol. 2013;14:627‐637. 23623280 10.1016/S1470-2045(13)70136-0

[9] Dutton SJ , Ferry DR , Blazeby JM , et al. Gefitinib for oesophageal cancer progressing after chemotherapy (COG): a phase 3, multicentre, double‐blind, placebo‐controlled randomised trial. Lancet Oncol. 2014;15:894‐904. 24950987 10.1016/S1470-2045(14)70024-5

[10] Zhou T , Fu H , Dong B , et al. HOXB7 mediates cisplatin resistance in esophageal squamous cell carcinoma through involvement of DNA damage repair. Thorac Cancer. 2019 10.1111/1759-7714.13142.[Epub ahead of print] . PMC7606015 31568655

[11] Ji Q , Chang L , Stanczyk FZ , et al. Impaired dihydrotestosterone catabolism in human prostate cancer: critical role of AKR1C2 as a pre‐receptor regulator of androgen receptor signaling. Cancer Res. 2007;67:1361‐1369. 17283174 10.1158/0008-5472.CAN-06-1593

[12] Zhang A , Zhang J , Plymate S , et al. Classical and non‐classical roles for pre‐receptor control of DHT metabolism in prostate cancer progression. Horm Cancer. 2016;7:104‐113. 26797685 10.1007/s12672-016-0250-9 PMC4859429

[13] Tai HL , Lin T‐S , Huang H‐H , et al. Overexpression of aldo‐keto reductase 1C2 as a high‐risk factor in bladder cancer. Oncol Rep. 2006;17:305‐311. 17203165

[14] Wang S , Yang Q , Fung KM , et al. AKR1C2 and AKR1C3 mediated prostaglandin D2 metabolism augments the PI3K/Akt proliferative signaling pathway in human prostate cancer cells. Mol Cell Endocrinol. 2008;289:60‐66. 18508192 10.1016/j.mce.2008.04.004

[15] Kazemi‐Noureini S , Colonna‐Romano S , Ziaee AA , et al. Differential gene expression between squamous cell carcinoma of esophageus and its normal epithelium; altered pattern of mal, akr1c2, and rab11a expression. World J Gastroenterol. 2004;10:1716‐1721. 15188492 10.3748/wjg.v10.i12.1716 PMC4572255

[16] Breton J , Gage MC , Hay AW , et al. Proteomic screening of a cell line model of esophageal carcinogenesis identifies cathepsin D and aldo‐keto reductase 1C2 and 1B10 dysregulation in Barrett’s esophagus and esophageal adenocarcinoma. J Proteome Res. 2007;7:1953‐1962. 10.1021/pr7007835 18396902

[17] Nancarrow DJ , Clouston AD , Smithers BM , et al. Whole genome expression array profiling highlights differences in mucosal defense genes in Barrett's esophagus and esophageal adenocarcinoma. PLoS One. 2011;6:e22513. 21829465 10.1371/journal.pone.0022513 PMC3145652

[18] Wang L‐S , Chow K‐C , Wu Y‐C , et al. Inverse expression of dihydrodiol dehydrogenase and glutathione‐S‐transferase in patients with esophageal squamous cell carcinoma. Int J Cancer. 2004;111:246‐251. 15197778 10.1002/ijc.11650

[19] Li C , Wu X , Zhang W , et al. High‐content functional screening ofAEG‐1andAKR1C2for the promotion of metastasis in liver cancer. J Biomol Screen. 2016;21:101‐107. 26318406 10.1177/1087057115603310

[20] Li C , Wu X , Zhang W , et al. AEG‐1 promotes metastasis through downstream AKR1C2 and NF1 in liver cancer. Oncol Res. 2015;22:203‐211. 10.3727/096504015X14386062091352 PMC7838427 26351209

[21] Chien CW , Ho IC , Lee TC . Induction of neoplastic transformation by ectopic expression of human aldo‐keto reductase 1C isoforms in NIH3T3 cells. Carcinogenesis. 2009;30:1813‐1820. 19696165 10.1093/carcin/bgp195

[22] Huang KH , Chiou SH , Chow KC , et al. Overexpression of aldo‐keto reductase 1C2 is associated with disease progression in patients with prostatic cancer. Histopathology. 2010;57:384‐394. 20840669 10.1111/j.1365-2559.2010.03647.x

[23] Ji Q , Aoyama C , Nien YD , et al. Selective loss of AKR1C1 and AKR1C2 in breast cancer and their potential effect on progesterone signaling. Cancer Res. 2004;64:7610‐7617. 15492289 10.1158/0008-5472.CAN-04-1608

[24] Jin YX , Zhou XF , Chen YY , et al. Up‐regulated AKR1C2 is correlated with favorable prognosis in thyroid carcinoma. J Cancer. 2019;10:3543‐3552. 31293659 10.7150/jca.28364 PMC6603404

[25] Wenners A , Hartmann F , Jochens A , et al. Stromal markers AKR1C1 and AKR1C2 are prognostic factors in primary human breast cancer. Int J Clin Oncol. 2016;21:548‐556. 26573806 10.1007/s10147-015-0924-2

[26] Sinreih M , Anko M , Zukunft S , et al. Important roles of the AKR1C2 and SRD5A1 enzymes in progesterone metabolism in endometrial cancer model cell lines. Chem Biol Interact. 2015;234:297‐308. 25463305 10.1016/j.cbi.2014.11.012

[27] Bortolozzi R , Bresolin S , Rampazzo E , et al. AKR1C enzymes sustain therapy resistance in paediatric T‐ALL. Br J Cancer. 2018;118:985‐994. 29515258 10.1038/s41416-018-0014-0 PMC5931104

[28] Gong YY , Jiang L , Li M , et al. Astrocyte elevated gene‐1 is a novel prognostic marker for breast cancer progression and overall patient survival. Clin Cancer Res. 2015;14:3319. 10.1158/1078-0432.CCR-07-4054 18519759

[29] Liu L , Lin C , Liang W , et al. TBL1XR1 promotes lymphangiogenesis and lymphatic metastasis in esophageal squamous cell carcinoma. Gut. 2015;64:26‐36. 24667177 10.1136/gutjnl-2013-306388

[30] Li H , Gao Q , Guo L , et al. The PTEN/PI3K/Akt pathway regulates stem‐like cells in primary esophageal carcinoma cells. Cancer Biol Ther. 2011;11:950‐958. 21467840 10.4161/cbt.11.11.15531

[31] Ma S , Bao JYJ , Kwan PS , et al. Identification of PTK6, via RNA sequencing analysis, as a suppressor of esophageal squamous cell carcinoma. Gastroenterology. 2012; 143:675‐686.e12. 22705009 10.1053/j.gastro.2012.06.007

[32] Sanchez‐Vega F , Mina M , Armenia J , et al. Oncogenic signaling pathways in the cancer genome atlas. Cell. 2018;173:321‐337.e10. 29625050 10.1016/j.cell.2018.03.035 PMC6070353

[33] Shirato A , Kikugawa T , Miura N , et al. Cisplatin resistance by induction of aldo‐keto reductase family 1 member C2 in human bladder cancer cells. Oncol Lett. 2014;7:674‐678. 24527071 10.3892/ol.2013.1768 PMC3919892

[34] He M , Li Q , Zou R , et al. Sorafenib plus hepatic arterial infusion of oxaliplatin, fluorouracil, and leucovorin vs sorafenib alone for hepatocellular carcinoma with portal vein invasion: a randomized clinical trial. JAMA Oncol. 2019;5:953‐960. 31070690 10.1001/jamaoncol.2019.0250 PMC6512278

[35] Wu MS , Wang GF , Zhao ZQ , et al. Smac mimetics in combination with TRAIL selectively target cancer stem cells in nasopharyngeal carcinoma. Mol Cancer Ther. 2013;12:1728‐1737. 23699656 10.1158/1535-7163.MCT-13-0017

[36] Kong N , Tao W , Ling X , et al. Synthetic mRNA nanoparticle‐mediated restoration of p53 tumor suppressor sensitizes p53‐deficient cancers to mTOR inhibition. Sci Transl Med. 2019;11:eaaw1565. 31852795 10.1126/scitranslmed.aaw1565 PMC7024563

[37] Rižner TL , Penning TM . Role of aldo–keto reductase family 1 (AKR1) enzymes in human steroid metabolism. Steroids. 2014;79:49‐63. 24189185 10.1016/j.steroids.2013.10.012 PMC3870468

[38] Zeng C‐M , Chang L‐L , Ying M‐D , et al. Aldo‐keto reductase AKR1C1–AKR1C4: functions, regulation, and intervention for anti‐cancer. Therapy Front Pharmacol. 2017;8:119. 28352233 10.3389/fphar.2017.00119 PMC5349110

[39] Shiiba M , Yamagami H , Yamamoto A , et al. Mefenamic acid enhances anticancer drug sensitivity via inhibition of aldo‐keto reductase 1C enzyme activity. Oncol Rep. 2017;37:2025‐2032. 28259989 10.3892/or.2017.5480

[40] Venema CM , Bense RD , Steenbruggen TG , et al. Consideration of breast cancer subtype in targeting the androgen receptor. Pharmacol Ther. 2019;200:135‐147. 31077689 10.1016/j.pharmthera.2019.05.005

[41] Salvi S , Bonafe M , Bravaccini S . Androgen receptor in breast cancer: a wolf in sheep’s clothing? A lesson from prostate cancer. Semin Cancer Biol. 2020;60:132‐137. 31002873 10.1016/j.semcancer.2019.04.002

[42] Li D , Zhou W , Pang J , et al. A magic drug target: androgen receptor. Med Res Rev. 2019;39:1485‐1514. 30569509 10.1002/med.21558

[43] Agapova OA , Malone PE , Hernandez MR . A neuroactive steroid 5alpha‐androstane‐3alpha,17beta‐diol regulates androgen receptor level in astrocytes. J Neurochem. 2006;98:355‐363. 16638015 10.1111/j.1471-4159.2006.03879.x

[44] Dozmorov MG , Yang Q , Matwalli A , et al. 5alpha‐androstane‐3alpha,17beta‐diol selectively activates the canonical PI3K/AKT pathway: a bioinformatics‐based evidence for androgen‐activated cytoplasmic signaling. Genomic Med. 2007;1:139‐146. 18923939 10.1007/s11568-008-9018-9 PMC2269037

[45] Ding J , Ning B , Gong W , et al. Cyclin D1 induction by benzo[a]pyrene‐7,8‐diol‐9,10‐epoxide via the phosphatidylinositol 3‐kinase/Akt/MAPK‐ and p70s6k‐dependent pathway promotes cell transformation and tumorigenesis. J Biol Chem. 2009;284:33311‐33319. 19801633 10.1074/jbc.M109.046417 PMC2785174

[46] Yang Q , Titus MA , Fung KM , et al. 5alpha‐androstane‐3alpha,17beta‐diol supports human prostate cancer cell survival and proliferation through androgen receptor‐independent signaling pathways: implication of androgen‐independent prostate cancer progression. J Cell Biochem. 2008;104:1612‐1624. 18320593 10.1002/jcb.21731

[47] Totani Y , Saito Y , Ishizaki T , et al. Leukotoxin and its diol induce neutrophil chemotaxis through signal transduction different from that of fMLP. Eur Respir J. 2000;15:75‐79. 10678624 10.1183/09031936.00.15107500

[48] Huang C , Li J , Song L , et al. Black raspberry extracts inhibit Benzo(a)Pyrene Diol‐Epoxide–Induced Activator Protein 1 activation and VEGF transcription by targeting the phosphotidylinositol 3‐kinase/Akt pathway. Cancer Res. 2006;66:581‐587. 16397275 10.1158/0008-5472.CAN-05-1951

[49] Li J , Tang MS , Liu B , et al. A critical role of PI‐3K/Akt/JNKs pathway in benzo[a]pyrene diol‐epoxide (B[a]PDE)‐induced AP‐1 transactivation in mouse epidermal Cl41 cells. Oncogene. 2004;23:3932‐3944. 15021902 10.1038/sj.onc.1207501

[50] Ainge GD , Parlane NA , Denis M , et al. Phosphatidylinositol mannosides: synthesis and adjuvant properties of phosphatidylinositol di‐ and tetramannosides. Bioorg Med Chem. 2006;14:7615‐7624. 16876422 10.1016/j.bmc.2006.07.003

[51] Xu Y , Lee SA , Kutateladze TG , et al. Chemical synthesis and molecular recognition of phosphatase‐resistant analogues of phosphatidylinositol‐3‐phosphate. J Am Chem Soc. 2006;128:885‐897. 16417379 10.1021/ja0554716 PMC2535791

[52] Eramo MJ , Mitchell CA . Regulation of PtdIns(3,4,5)P3/Akt signalling by inositol polyphosphate 5‐phosphatases. Biochem Soc Trans. 2016;44:240‐252. 26862211 10.1042/BST20150214

