# Supplementary material for: AKR1C2 acts as a targetable oncogene in esophageal squamous cell carcinoma via activating PI3K/AKT signaling pathway
Source: J Cell Mol Med. 2020 Jul 17;24(17):9999–10012. doi: 10.1111/jcmm.15604 (PMC7520259; doi:10.1111/jcmm.15604)
Supplement: Supplementary file 1 — Appendix S1 [file JCMM-24-9999-s001.docx]

**Appendix S1**

**Supplementary materials and methods**

**Data acquisition of public databases**

Some data about the mRNA expression of AKR1C2 in esophageal tissues were obtained from public databases TCGA (<http://ualcan.path.uab.edu/index.html>) and Oncomine (https://www.oncomine.org/). The data from TCGA includes normal tissues (n=11), adenocarcinoma tissues (n=89), squamous cell carcinoma tissues (n=95), while the Oncomine data was ESCC tissues and corresponding nontumor tissues from Hu team (n=17) and Su team (n=53).

**RNA-sequencing and bioinformatic analysis**

RNA-sequencing was performed on three pairs of matched ESCC tumor and adjacent normal tissues (one female and two male) using the Illumina HiseqXTEN platform at GenanBio Co., Ltd (Guangzhou, China). The raw data was normalized and z-score-transformed by R software. The RNA-sequencing analysis was also performed for knockdown, overexpression and control treated ESCC cell lines using the Illumina Novaseq™ 6000 platform at LC-tech, Ltd (Hangzhou, China). Followed by GO and KEGG analysis.

**MTS assay**

Cells were seeded in 96-well plates at an initial density of 1000 cells/well, with 6 repetitions per group. The wells without cells were used as blank control. At each corresponding time points after seeding (1 to 6 days), MTS (Cell Titer 96 Aqueous One Solution Cell Proliferation Assay solution, Promega, Wisconsin, USA) and DMEM were mixed at a ratio 1:10, 200 μL of the mixture was added to each well and incubated for 3 h. Their absorbance value was detected at 490nm by microplate research reader (Bio-Tek EPOCH2, VT, USA).

**Colony formation assays**

Cells (1000 per well) were seeded into a 6-well plate with 2 mL cultured medium which was changed every 3 days. Two weeks after plating, the cells were washed with PBS, fixed with 10% formaldehyde for 15 min, stained with 5% crystal violet for 30 min at room temperature before counting the number of colonies formed.

**Wound-healing assay**

A wound-healing assay was performed in cells transiently transfected with siRNA or overexpression plasmid. The cells were seeded in 6-well plates (1x10^6^ cells per well) and when grown to full confluent density, a crossing scrape was made in each well using a sterile 20 μL pipette tip. Any detached cells were washed away by PBS. Images were taken using an inverted microscope (OLYMPUS IX73, Tokyo, Japan) at 0 hours, 24 hours and 36 hours at the same fields.

**Migration assay**

Migration assays were performed using chambers (Corning, Michigan, USA) based on the production instruction. Fifty thousand cells in serum-free DMEM were seeded in the upper chamber. The bottom chamber contained DMEM with 10% FBS. After incubation for 24 hours, fixed with methanol for 15 min and stained with 5% crystal violet for 30 min at room temperature, the cells in the upper surface of the membranes were removed by a cotton swab. The lower surface of the membranes were observed under a microscope (NIKON ECLIPSE 80i, Tokyo, Japan) at 100 magnification in four randomly selected fields per well for any migrated cells.

**Fluorescence activated cell sorting assay**

Cell apoptosis assays were detected by using the PI/Annexin V FITC Apop Dtec Kit (BD, New Jersey, USA) according to the manufacturer’s manual. Briefly, when the cells were grown to ~60% confluency, they were incubated with or without drug for 72 hours. Cells were collected and processed for analysis using a flow cytometer (Beckman, CA, USA).

**IC50 determination of cisplatin**

The IC50 of cisplatin (Sigma, Shanghai, China) was tested by dose-dependent toxicity assay in several ESCC parental cell lines (KYSE30, KYSE180, KYSE410, KYSE510, KYSE520, EC109) and gene manipulated cells (vector-KYSE30, AKR1C2-KYSE30; si#scramble-KYSE180, si#1-KYSE180), after 72 hours incubation with cisplatin, cell viability was determined by MTS assay.

**Cisplatin, LY294002 single drug or combination therapy**

The indicated cells were treated with cisplatin (Sigma, Shanghai, China), LY294002 (Selleck, Shanghai, China) single drug or cisplatin plus LY294002. The concentration of cisplatin was lower than its IC50, and the concentration of LY294002 was 5 μM or 10 μM. After drug exposure for 72 hours, MTS, Western blotting and cell apoptosis assays were performed.

**Ursodeoxycholic acid (UDCA) inhibition of AKR1C2 enzyme activity assay**

Indicated cells were treated with 10 μM UDCA (Sigma, Shanghai, China) for 24 hours, followed by MTS, transwell assays, but incubated 72 hours for Western blotting experiments.

**Cisplatin, UDCA single drug or combination therapy**

For drug combination therapy assays, UDCA alone or combined with cisplatin were added to the indicated cells for 72 hours, the concentration of DUCA was 5 μM or 10 μM, and the cisplatin concentration was 5μg/mL, then, the cell viability was detected by MTS assays.

**Animal experiment**

Three-week-old male BALB/c mice were purchased from the Guangdong Medical Laboratory Animal Center (Guangzhou, China). All animal study protocols were approved by the Research Animal Resource Center of Sun Yat-Sen University. The mice were subcutaneously injected with 5×10^6^ cells with AKR1C2 silence or overexpression. The tumors formed by ESCC cells were measured every three days with calipers and calculated using the following formula: Volume (mm^3^) = [length (mm) × width^2^ (mm^2^)]/2. At the end of the experiment, the mice were euthanized and their tumors were dissected. For lung metastasis experiment, 200 μL of cultured medium containing 1×10^5^ cells were injected into the tail vein of the mice. After 12 weeks, the mice were euthanized. The lungs were weighed, then fixed with picric acid and stained with hematoxylin and eosin (HE). The lung metastasis numbers were counted under high magnification microscope (NIKON ECLIPSE 80i, Tokyo, Japan) fields. In the drug-treated nude mice models, KYSE180 cells (5x10^6^) were subcutaneously injected into the right flank of 20 male mice, when the tumor volume was about 100 mm^3^, the mice were randomly divided into five groups that were untreated, treated with cisplatin, LY294002, or both by intraperitoneal (i.p.) injection every five days and for three cycles, the cisplatin concentration was 5 mg/kg, the concentration of LY294002 was 50 mg/kg or 100 mg/kg. At 45 days after injection, mice were euthanized and excised the tumor for IHC.
