# Supplementary material for: AKR1C2 acts as a targetable oncogene in esophageal squamous cell carcinoma via activating PI3K/AKT signaling pathway
Source: J Cell Mol Med. 2020 Jul 17;24(17):9999–10012. doi: 10.1111/jcmm.15604 (PMC7520259; doi:10.1111/jcmm.15604)
Supplement: Supplementary file 2 — Table S1‐S4 [file JCMM-24-9999-s002.docx]

**Table S1 Primers used for real-time PCR.**

| primers | sequence (5' to 3') |
| --- | --- |
| AKR1C2-F | AAGTAAAGCTCTAGAGGCCGT |
| AKR1C2-R | CTCTGGTCGATGGGAATTGCT |
| GAPDH-F | GTCTCCTCTGACTTCAACAGCG |
| GAPDH-R | ACCACCCTGTTGCTGTAGCCAA |

**Table S2 Clinical characteristics of the training cohort.**

| **Characteristics** | **No. of cases(%)** |
| --- | --- |
| **Age†，years** | |
| <60 | 22（46.81） |
| ≥60 | 25（53.19） |
| **Gender** |  |
| Male | 34（72.34） |
| Female | 13（27.66） |
| **Smoking status** | |
| yes | 27（57.45） |
| no | 20（42.55） |
| **Alcohol consumption** | |
| yes | 13（27.66） |
| no | 34（72.34） |
| **Family history** | |
| yes | 4（8.51） |
| no | 43（91.49） |
| **Tumor location** | |
| upper | 2（8.51） |
| middle | 23（48.94） |
| lower | 20（42.55） |
| **Differentiation** | |
| well | 6（12.77） |
| moderate | 28（59.57） |
| poor | 13（27.66） |
| **pT status** | |
| T1 | 9（19.15） |
| T2 | 11（23.40） |
| T3 | 22（46.81） |
| T4 | 5（10.64） |
| **pN status** | |
| N0 | 32（68.09） |
| N1 | 10（21.28） |
| N2 | 3（6.38） |
| N3 | 2（4.26） |
| **Pathological stage** | |
| Ⅰ | 11（23.40） |
| Ⅱ | 21（44.68） |
| Ⅲ | 14（29.79） |
| Ⅳ | 1（2.13） |
| **Survival status** | |
| alive | 23（48.94） |
| dead | 24（51.06） |

**^†^** Median age.

**Table S3 Sequences of the siRNAs and shRNA against AKR1C2.**

|  | sequence (5' to 3') |
| --- | --- |
| Scramble-sense | UUCUCCGAACGUGUCACGUTT |
| Scramble-antisense | ACGUGACACGUUCGGAGAATT |
| AKR1C2-si#1-sense | GGCCGUCAAAUUGGCAAUATT |
| AKR1C2-si#1-antisense | UAUUGCCAAUUUGACGGCCTT |
| AKR1C2-si#2-sense | CCAUAUUGAUUCUGCACAUTT |
| AKR1C2-si#2-antisense | AUGUGCAGAAUCAAUAUGGTT |
| AKR1C2-sh#1 | CCACCATATTGATTCTGCACA |

**Table S4 Sequences of the AKR1C2 overexpression plasmid.**

| AGATCCGCTAGCGCTACCGGACTCAGATCTCGAGCGCCACCATGGATTCGAAATACCAGTGTGTGAAGCTGAATGATGGTCACTTCATGCCTGTCCTGGGATTTGGCACCTATGCGCCTGCAGAGGTTCCTAAAAGTAAAGCTCTAGAGGCCGTCAAATTGGCAATAGAAGCCGGGTTCCACCATATTGATTCTGCACATGTTTACAATAATGAGGAGCAGGTTGGACTGGCCATCCGAAGCAAGATTGCAGATGGCAGTGTGAAGAGAGAAGACATATTCTACACTTCAAAGCTTTGGAGCAATTCCCATCGACCAGAGTTGGTCCGACCAGCCTTGGAAAGGTCACTGAAAAATCTTCAATTGGACTATGTTGACCTCTATCTTATTCATTTTCCAGTGTCTGTAAAGGAGGACATAGGGATTTTAACATGGAAGAAGAGCCCTAAACATAACTCCTAAGAATTCTGCAGTCGACGGTACCGCGGGCCCGGGATCCGCCCCTCTCCCTCCCCCCCCCCTAACGTTACTGGCCGAAGCCGCTTGGAATAAGGCCGGTGTGCGTTTGTCTATATGTTATTTTCCACCATATTGCCGTCTTTTGGCAATGTGAGGG |
| --- |
