# Supplementary material for: AKR1C2 acts as a targetable oncogene in esophageal squamous cell carcinoma via activating PI3K/AKT signaling pathway
Source: J Cell Mol Med. 2020 Jul 17;24(17):9999–10012. doi: 10.1111/jcmm.15604 (PMC7520259; doi:10.1111/jcmm.15604)

**Supplementary Legends and Figures**

**Figure S1. AKR1C2 expression was upregulated in ESCC.** **(A)** AKR1C2 was significantly upregulated in ESCC tissues (n=95) compared with normal tissues (n=11) and adenocarcinoma tissues (n=89) in the TCGA database. **(B, C)** AKR1C2 expression was higher in ESCC relative to matched normal tissues in Hu Esophagus Statistics (n=17) and Su Esophagus Statistics (n=53) in the Oncomine database. *** *p* < 0.001, NS, not significant. Student t test.

**Figure S2. Validation of gene manipulation in cell lines.** **(A, B)** Decreased expression of AKR1C2 were confirmed by RT-qPCR in KYSE410 and EC109 cells after AKR1C2 knockdown**. (C)** Overexpression of AKR1C2 were confirmed by RT-qPCR in KYSE30 cell after transfection of AKR1C2 plasmid. ***p* < 0.01, *** *p* < 0.001. Student t test.

**Figure S3. LY294002 can reverse the EMT phenomenon induced by AKR1C2.** Levels of pAKT, E-cadherin, Vimentin were determined by Western blotting analysis after vehicle (DMSO) or LY294002 (10 μM) was added to the indicated cells and incubated for 72 hours. LY, LY294002.

**Figure S4. The combination therapy of cisplatin and LY294002 has synergistic antitumor effects. (A)** MTS assays were performed to detected cells viability after cisplatin, LY294002 single or combinational use in indicated cells for 72 hours. ***p* < 0.01, ****p* < 0.001, NS, not significant. Student t test. **(B)** At 72 hours after drug exposure, KYSE30^L^ and KYSE180H were double stained with propidium iodide (PI) and Annexin V-FITC, and analyzed by flow cytometry. Superscript “L” and “H” represent low expression and high expression respectively.

**Figure S5. AKR1C2 may involves in the biosynthesis of inositol and steroids, and its Enzyme activity inhibitor can reverse EMT. (A)** KYSE30 cell was transfected with empty vector or AKR1C2 plasmid, GO analysis was conducted after RNA-sequencing. **(B)** EC109 cell was transfected with AKR1C2 siRNA or scrambled siRNA, KEGG pathways were showed after RNA-sequencing. The steroid hormone biosynthesis and steroid biosynthesis were indicated by rectangle. **(C)** The expression levels of pAKT, E-cadherin, and Vimentin were detected by Western blotting after the indicated cells were incubated with UDCA for 72 hours.


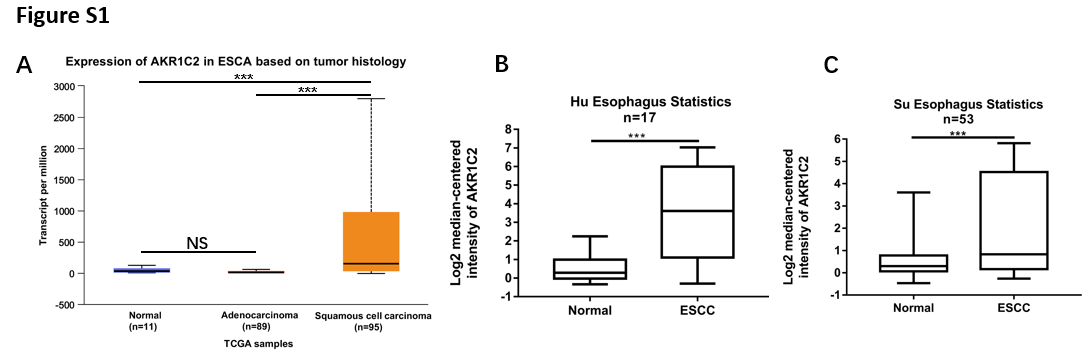


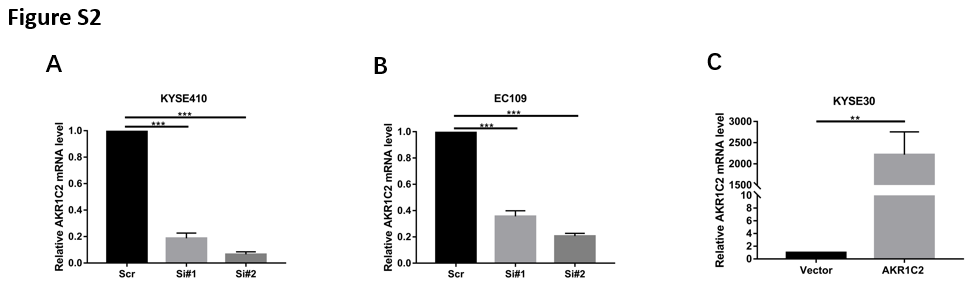


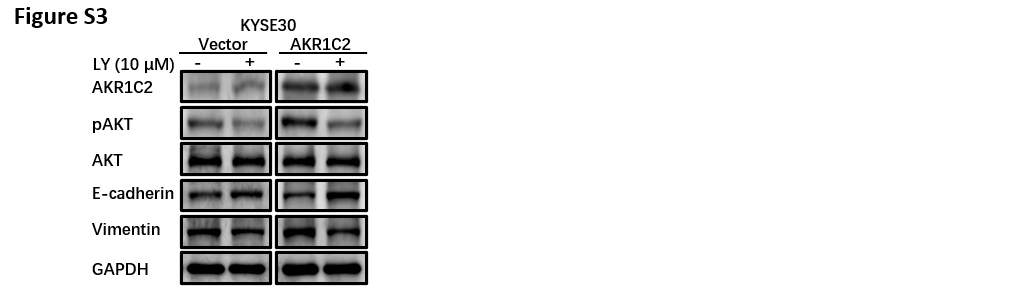


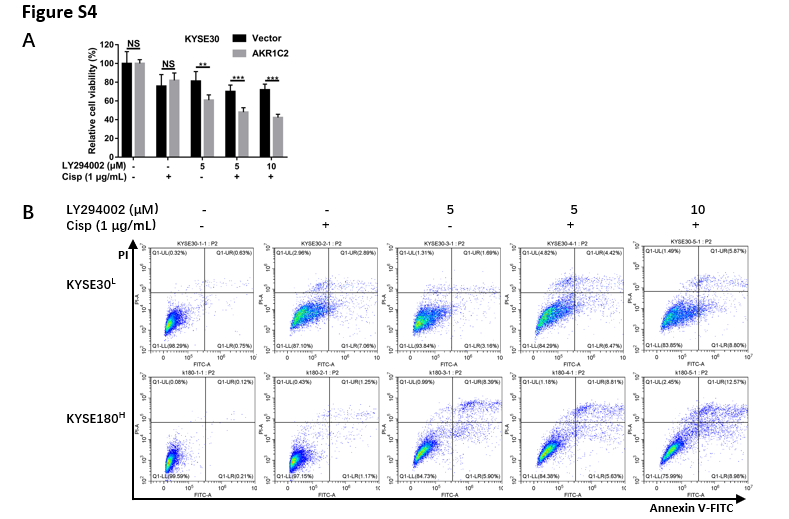


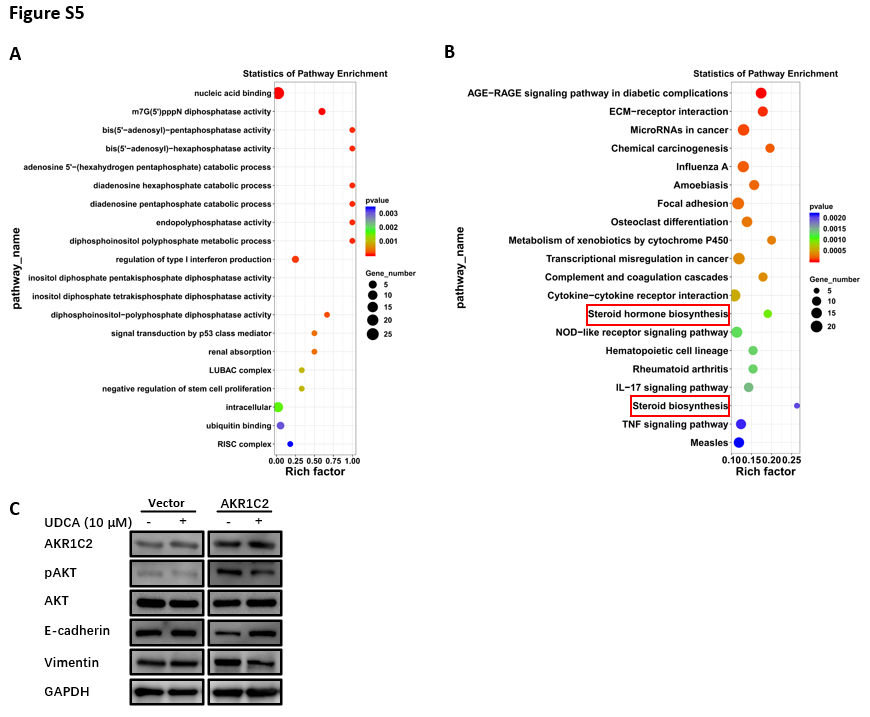

Supplement: Supplementary file 3 — Fig S1‐S5 [file JCMM-24-9999-s003.docx]
